# Encephalitis Followed by Optic Neuritis: A Case Report and Review of Literature

**DOI:** 10.12669/pjms.293.3475

**Published:** 2013

**Authors:** Shariful Hasan, Hamidon B. Basri, Lim P. Hin, Johnson Stanslas

**Affiliations:** 1 Shariful Hasan, MBBS, FCNp, Neurology Unit, Institute of Gerontology, Department of Medicine, Faculty of Medicine and Health Sciences, University Putra Malaysia, Malaysia.; 2 Hamidon B. Basri, MD, MMed, Neurology Unit, Department of Medicine, Faculty of Medicine and Health Sciences, University Putra Malaysia, Malaysia.; 3 Lim P. Hin, MD, ABPN (Dip), Neurology Unit, Department of Medicine, Faculty of Medicine and Health Sciences, University Putra Malaysia, Malaysia.; 4 Johnson Stanslas, MSc, PhD, Pharmacotherapeutics Unit, Department of Medicine, Faculty of Medicine and Health Sciences, University Putra Malaysia, Malaysia.

**Keywords:** Viral encephalitis, Herpes simplex virus, Optic neuritis

## Abstract

Encephalitis has been included in the causes of optic neuritis, but post encephalitic optic neuritis has been rarely reported. Majority of the cases of optic neuritis are either idiopathic or associated with multiple sclerosis, especially in western countries. This is very important in the Asian population where the incidence and prevalence of multiple sclerosis is not as high as in the Western countries. Although post infectious optic neuritis is more common in children, it can also be found in adults and is usually seen one to three weeks after a symptomatic infective prodrome. Here, we present a case of a 48 year-old-male who developed optic neuritis following viral encephalitis. His first presentation was with severe headache of two weeks duration. Viral encephalitis was diagnosed and treated. The patient presented again three weeks later with right eye pain and other features typical of optic neuritis. Corticosteroid therapy facilitated prompt recovery. Optic neuritis is an uncommon manifestation of encephalitis. It is important that both doctors and patients remain aware of post infectious cause of optic neuritis, which would enable a timely diagnosis and treatment of this reversible cause of vision loss.

## INTRODUCTION

Optic neuritis is defined as the inflammation of the optic nerve, which causes sudden painful vision loss or reduced vision in the affected eye.^1^^-^^3^ Although most cases of optic neuritis are either idiopathic or associated with multiple sclerosis, post infectious cause is also known. This is of particular importance in the Asian population where the incidence and prevalence of multiple sclerosis is not as high as in the Western countries. While post infectious optic neuritis is more common in children, it can also be found in adults. It is usually seen one to three weeks after a symptomatic infective prodrome.^3^^-^^5^ The objective of presenting this case report is to increase awareness of the doctors and patients on post infectious optic neuritis, which is rare, but a reversible cause of vision loss if diagnosed and treated promptly.

## CASE PRESENTATION

A 48-year-old male presented with complaints of severe headache of two-week duration. The headache was in the right parietal region accompanied by nausea and vomiting. There was neither any history of fever, photophobia, blurring of vision or diplopia, nor any significant past medical history. He was previously seen in another hospital; the brain Computed Tomography (CT) scan was reported to be normal. 

On physical examination, the vital signs were within normal limits, and the patient was afebrile. Mental status examination did not reveal any abnormality. On clinical examination of the motor system, the tone and power in all the four limbs were normal with exaggerated (3+) reflexes. There was no neck stiffness, and Kernig’s sign was negative. Gait and coordination were normal. The sensory system and the cranial nerves were normal. Fundoscopy did not reveal any abnormality. No significant finding was revealed in other systemic examinations.

**Fig.1:**
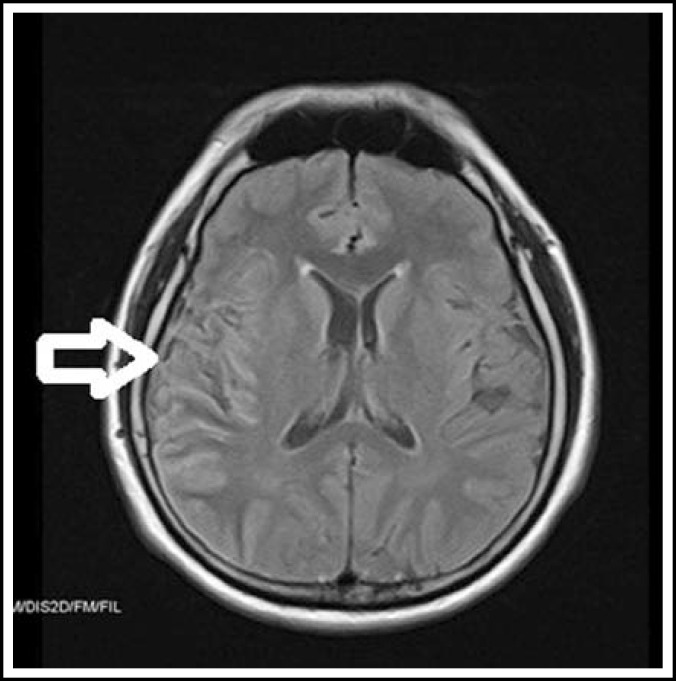
MRI brain before treatment showing bright T2/FLAIR cortical images at the right parietal and temporal region

Routine full blood count and biochemistry did not show any abnormality. However, enzyme-linked immunosorbent assay (ELISA) of the patient’s serum was positive for anti- herpes simplex virus (HSV) IgM antibody. Lumbar puncture was not carried out since the patient refused it. The electroencephalogram (EEG) did not reveal any abnormality. The Magnetic Resonance Imaging (MRI) brain revealed bright T2/FLAIR cortical images at right parietal and temporal region (Fig.1).

**Fig.2:**
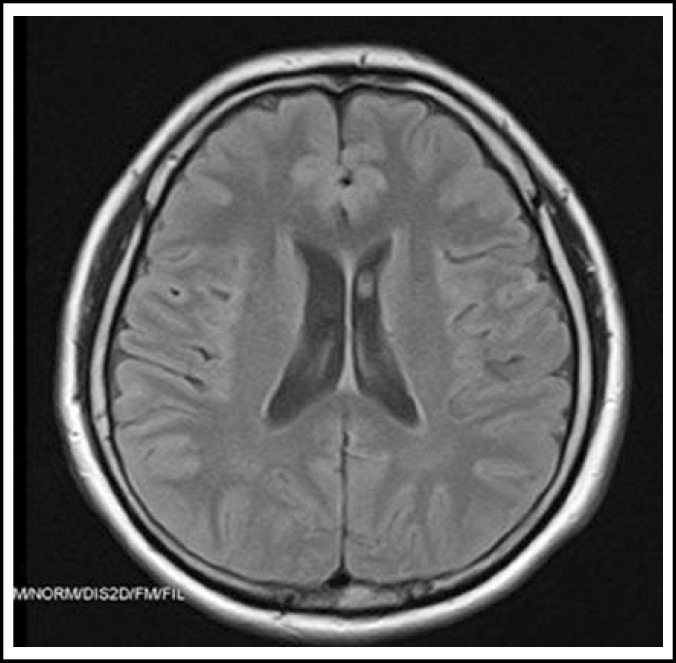
MRI one week following treatment clearly showing marked reduction of cortical enhancement

A presumptive diagnosis of herpes simplex encephalitis (HSE) was made on the basis of serological and MRI findings; empirical treatment with intravenous acyclovir 750 mg eight hourly was initiated. He also received hydrocortisone 8 mg intravenous followed by oral prednisolone 40 mg daily. The headache was relieved, and the patient had no complaint while in the ward. A repeat brain MRI, which was done one week later, showed that the cortical enhancement has markedly reduced indicating response to treatment (Fig.2). On discharge the patient was advised to repeat MRI and EEG after one week. 

Three weeks after the initial admission, the patient presented again with complaints of right retro-orbital pain of two days duration. The pain was accentuated with eye movements. The patient also noticed that he could not see clearly with the affected eye. Clinical examination revealed red colour desaturation and reduced visual acuity. Ophthalmoscopic examination showed a swollen right optic disc. Visual evoked potential (VEP) revealed small amplitude and delay in the right P100 latency (right: 122.40 ms, left: 113.10 ms). Right optic neuritis was diagnosed, which was treated with intravenous methylprednisolone 250 mg six hourly. The right eye pain was relieved, and the vision gradually improved. A diagnosis of post encephalitic right optic neuritis was made. 

## DISCUSSION

Viral encephalitis can be caused by a variety of pathogens; however, the most important causative agent is HSV. HSE has gained importance not only for being the most common encephalitis encountered, but also for its severity and serious neurologic sequelae. While any delay in treatment is associated with high morbidity and mortality, early diagnosis and prompt treatment with intravenous acyclovir is associated with favourable outcome in most of the cases. Therefore, early diagnosis and treatment is of utmost importance. However, diagnosis of HSE is not always easy as it can frequently present with atypical features, and imaging done early in the course of the disease can at times be normal. Moreover, sometimes MRI may show a completely extra-temporal involvement (temporal involvement is typically seen in HSE), which adds further difficulty to diagnosis. Hence, early treatment of suspected viral encephalitis with intravenous acyclovir even before a definitive diagnosis can be made is now an accepted practice.^6^^-^^8^

In the current case, a definitive diagnosis of HSE was not possible since the patient refused lumbar puncture and therefore, cerebrospinal fluid polymerase chain reaction (CSF PCR) to identify the pathogen could not be done. We made a presumptive diagnosis based on the presence of anti-HSV IgM antibody in the serum and MRI picture. The resolution of the lesion in the subsequent MRI following treatment with acyclovir further supported the diagnosis. The CT scan is less sensitive and does not show any abnormality in about 33% of the cases, which could be the reason for a normal CT scan in our patient.^8^

Although the prognosis of HSE has considerably improved since the advent of specific treatment with intravenous acyclovir, complications are known to occur. While complications like cognitive impairment, neuropsychological dysfunctions, seizures, movement disorders including dystonias, and parkinsonism have been observed, optic neuritis is rarely seen.^8^ Even though viral encephalitis is included in the causes of post infectious optic neuritis, not many cases have been reported.

Post infectious optic neuritis is usually bilateral and is more common in children.^5^^,^^9^ This explains the reason why most of the reported cases of post infectious optic neuritis are from the paediatric population; usually following infections like mumps, measles, chicken pox, which are more common in children. Post vaccination optic neuritis has also been described.^10^^-^^12^ A case of optic neuritis following rubella encephalitis in a five-year-old boy has been presented by Yoshida et al^13^ twenty years ago. A few cases in adults have also been reported. Azevedo et al^14^ and Galbussera et al^15^ reported cases of optic neuritis following chicken pox in adults. Ihanamaki et al^16^ reported a case of parainfectious bilateral optic neuritis associated with echoviral meningitis in an adult patient.

Here, we describe a case of optic neuritis following viral encephalitis in a 48-year-old man. The typical triad of optic neuritis which includes sub-acute unilateral loss of vision, periocular pain and impaired colour vision^9^ were seen in our patient presenting approximately three weeks after initial hospitalisation for encephalitis. Optic nerve involvement three weeks after the initial viral infection suggests a delayed immune response. Prompt treatment with intravenous corticosteroids relieved the symptoms. The Optic Neuritis Treatment Trial (ONTT), and various other clinical trials, which have been done to investigate the treatment of optic neuritis have found that corticosteroids definitely accelerate the recovery even though they may not affect the final visual outcome to a great extent. Post infectious or parainfectious optic neuritis is known to have a favourable prognosis as compared to other varieties.^3^^,^^9^^,^^17^^,^^18^ The delayed onset and the favourable recovery both support an autoimmune process in the current case. It is imperative that we remain aware of post infectious cause of optic neuritis, which would enable a timely diagnosis and treatment. Although optic neuritis is an uncommon manifestation of encephalitis, we suggest ophthalmologic examination including fundoscopy should be routinely performed during follow-up visit of a patient with viral encephalitis.

***Limitation of the study: ***In this case, the patient refused lumbar puncture, which is very common in Malaysia due to superstitious belief of being paralyzed by the procedure. Therefore, cerebrospinal fluid (CSF) cytology for differentiating the causes of encephalitis, and CSF PCR to identify the pathogen could not be done.

## CONCLUSION

Viral encephalitis is associated with high morbidity and mortality, especially if there is any delay in treatment. Empirical treatment for viral encephalitis with intravenous acyclovir should be carried out in all suspected cases even before accurate diagnosis can be made. Optic neuritis can occur following viral encephalitis, and awareness regarding this is essential for both doctors and patients, which would enable a timely diagnosis and treatment of this reversible cause of vision loss. The need of this awareness is especially important in countries like Malaysia, where lumber puncture cannot be performed as a routine procedure for neurological disorders.

***Consent*** **:** Informed written consent was obtained from the patient for publication of this case report and any accompanying images

## Authors Contribution

We certify that we participated sufficiently in the intellectual content, conception and design of this work and the analysis and interpretation of the data (when applicable), as well as writing of the manuscript.
